# Oral and anal sexual practice and associated factors among preparatory school youths in Dire Dawa city administration, Eastern Ethiopia

**DOI:** 10.1371/journal.pone.0206546

**Published:** 2018-11-07

**Authors:** Mengistu Tiruneh Alemu, Yadeta Dessie, Tesfaye Gobena, Yohannes Teka Mazeingia, Abdu Oumer Abdu

**Affiliations:** 1 MPH in Reproductive Health, Direct Support professional at Key Stone Human Services, Harrisburg, Pennsylvania, United States of America; 2 Department of Public Health, Haramaya University, Harar, Ethiopia; 3 Department of Public Health, Debre Markos University, Debre Markos, Ethiopia; 4 Department of Health and Medicine, Wolkite University, Wolkitie, Ethiopia; University of Gondar, ETHIOPIA

## Abstract

**Background:**

Human Immunodeficiency Virus greatly affects young peoples in developing countries, of which sexual transmission is the major route including vaginal, oral and anal sex. Understanding the full range of sexual behavior among young people, especially oral and anal sexual experience is very crucial to design appropriate intervention strategies. This study was to assess oral and anal sexual experience and associated factors among preparatory school youths in Dire Dawa city, Eastern Ethiopia.

**Methods:**

School based cross sectional study was conducted among 1067 school youths attending preparatory school. Systematic random sampling method was used to select study participants. Data were collected using self-administered questionnaire and entered in to Epi-data version 3.3.1 and exported to SPSS 20 for analysis. Descriptive and bivariate logistic regression was done. Variables in bivariate analysis with P< 0.25 were entered to multiple bivariate logistic regression analysis to determine predictor variables. P < 0.05 was declared as statistically significance and AOR with 95% CI used to assess strength of association.

**Results:**

The proportion of youths who reported having oral sex was 9% (88) and that of anal sex was 6.7% (66). Having multiple sexual partners was reported by 65.8% and 56.5% of youths who ever engaged in oral and anal sex respectively. From those who ever engaged to oral sex and anal sex 15.9% and 34.8% consistently used condom respectively. Oral sex practice was significantly associated with intimate partner ever engaged to oral sex practice (AOR = 4.53, 95% CI: 2.26–9.05), ever engaged to vaginal sex (AOR = 16.38, 95% CI: 7.22–37.19), older age (20-24years) (AOR = 2.45, 95% CI: 1.24–4.86), ever drinking alcohol (AOR = 2.11,95% CI:1.02–4.34), and ever smoke shisha (AOR = 2.85,95% CI:1.4–5.83). Similarly anal sex experience was significantly associated with intimate partner ever engaged to anal sex (AOR = 5.34, 95% CI: 4.2–26.98), ever engaged to vaginal sex (AOR = 10.64, 95% CI: 2.39–11.9), ever watching pornographic movies (AOR = 3.86, 95% CI: 1.45–10.29) and parental monitoring of youth’s sexual behavior (AOR = 2.63, 95% CI: 1.12–6.19).

**Conclusions:**

Significant proportion of youths had engaged in oral and anal sexual practice and multiple sexual partners were common among youths for oral and anal sex. In the contrary consistent condom use was very poor. A combination of sexual health education intervention strategies should be implemented at family, school and community level.

## Background

Sexual practice has different forms including oral sex, vaginal sex, anal sex and masturbation. Significant proportion of youths engaged in these sexual behaviors in varied level [1, 2]. Youths who practiced risk sexual behaviors suffered disproportionately from negative sexual and reproductive health outcomes including HIV [3, 4]. Sexual transmission acknowledged as the main route of HIV transmission among youths [5]. Moreover, from sexual HIV transmission risks, unprotected receptive anal sexual practice carried the highest risk of HIV transmission when it is 20 times than risk of HIV acquisition through unprotected receptive vaginal intercourse (1.7% vs. 0.08%) [6]. But the risk of HIV transmission for insertive vaginal and insertive anal practice is almost similar. Oral sex assumed to have low risk for HIV transmission as compared to other routs but it can also transmit HIV for both insertive and respective one [7].

From demographic perspective, young people accounted significant proportion of the population worldwide. Half of the world’s population is under 25 years old and young people age 10–24 years constitute about a third of the Sub-Saharan Africa (SSA) population [8], meaning that some three and half billion children and young people are, or will soon be, of reproductive age. Youth age 15–24 years account 18.2% of Ethiopia total population based on the 2014 mini Ethiopian demographic and health survey (EDHS) report [9]

Though different HIV prevention and control intervention implemented at global and nation level to protect youths from adverse sexual and reproductive health outcomes, significant proportion of youths people are still being affected with STIs including HIV/AIDS. There were an estimated 250 000 new HIV infections among adolescents in 2013 [10]. Female youths were the most affected with HIV and the prevalence of HIV among them is about 15% total HIV burden especially in Sub Saharan Africa (SSA) countries [10 The burden of HIV among youths in Ethiopia was estimated to be 0.3% [11]. By the year 2013 from HIV infected adolescents at global level 7% of them resided in Ethiopia [10].

Studies in other parts of the world showed that youths practiced oral sex more than vaginal sex for several reasons [12, 13]. Beside various researches showed significant proportion of youths practiced anal sex at global level [14]. Though there were numerous studies conducted sexual and reproductive issues of youths in Ethiopia [15–17] it is rare to observe when they assessed youth’s anal and oral sexual experience and their predictors. As only study conducted to assess anal and oral sexual behavior of youths in Ethiopia discussed 5.4% and 4.3% of in-school youths in the Addis Ababa ever practiced oral sex and anal sex respectively [1]. It is not only researchers who failed to address anal sexual behavior of youths but the national government and other stakeholders who engaged in youth related issues did not offer anal sexual behavior related issues. So this study helps to describe the level and determinants of oral and anal sex practice among school youths in Dire Dawa city administration so as to design appropriate intervention strategies on sexual behavior of in school youths at national level.

## Methods

### Study area and period

The study was conducted in Dire Dawa city administration among preparatory school youths, which is found in Eastern part of Ethiopia 515kms from Addis Ababa [18]. Dire Dawa has a total population of 341,834, of whom 171,461 are men and 170,461 women; 233,224 or 68.23% of the population are urban inhabitants [19]. There are 20 secondary schools, 103 primary schools, 88 KG and 8 preparatory schools in the region (4 private and 4 governmental). A total of 3147 were preparatory school students were registered as of 2014 [20]. The study was conducted from March 1–10, 2016.

### Study design

School based cross sectional study was conducted among randomly selected youth students in preparatory schools in Dire Dawa city administration in 2015/2016. Thinking sensitivity of sexual behavior particularly anal and oral sexual behavior from Ethiopia cultural perspective, we believed collecting data through interview third party might break confidentiality issues. Even they might not provide correct information about their sexual experience. Therefore, those who were unable to fill the questionnaire without assistance were excluded.

### Sample size determination

We calculated sample size for each specific objectives and the maximum sample size we got from calculated samples taken as final sample size of the study. We get maximum sample size with objective we set to identify determinants of oral and anal sex. Variables which were significantly associated with the outcome variables (oral and anal sex experience) in other studies were considered taken to calculate the sample size. Open Epi save software program was used to simplify sample calculation and we used confidence level of 95%, margin of error of 5% and power of 80% during sample size calculation. The possible calculated sample size is shown in Table 1 and adds an additional 10 percent contingency for non-response. Taking school youths, age as determinant for anal sex practice, using computer software found minimum sample size of 711. Considering rare nature of event and design effect (1.5), the final sample size was 1067 [1]

**Table 1:** Sample size determination for oral and anal sexual experience and predictor of oral and anal sex among preparatory school students of Dire Dawa city administration, Ethiopia. 2016.

The 2^nd^ specific objective	Factor considered	Proportion value	Sample size
Factors associated with oral and anal sex experience	Maternal educational status[1]	% of youths engaged anal sex whose mother was illiterate or no formal education = 16.4% % of youths engaged anal sex whose mother has a formal education = 1.5%	154
	Age[1]	% of youths experience oral sex who are young adolescents (15–16) = 9.9% % of youths experience oral sex who are older adolescents (17 and above) = 4%	711
	Ever experience sexual intercourse [21]	% of youths experience oral sex who ever engaged in sexual intercourse = 66.6% % of youths experience oral sex and who never engaged in sexual intercourse = 16%	40

### Sampling procedure

**Fig 1:**
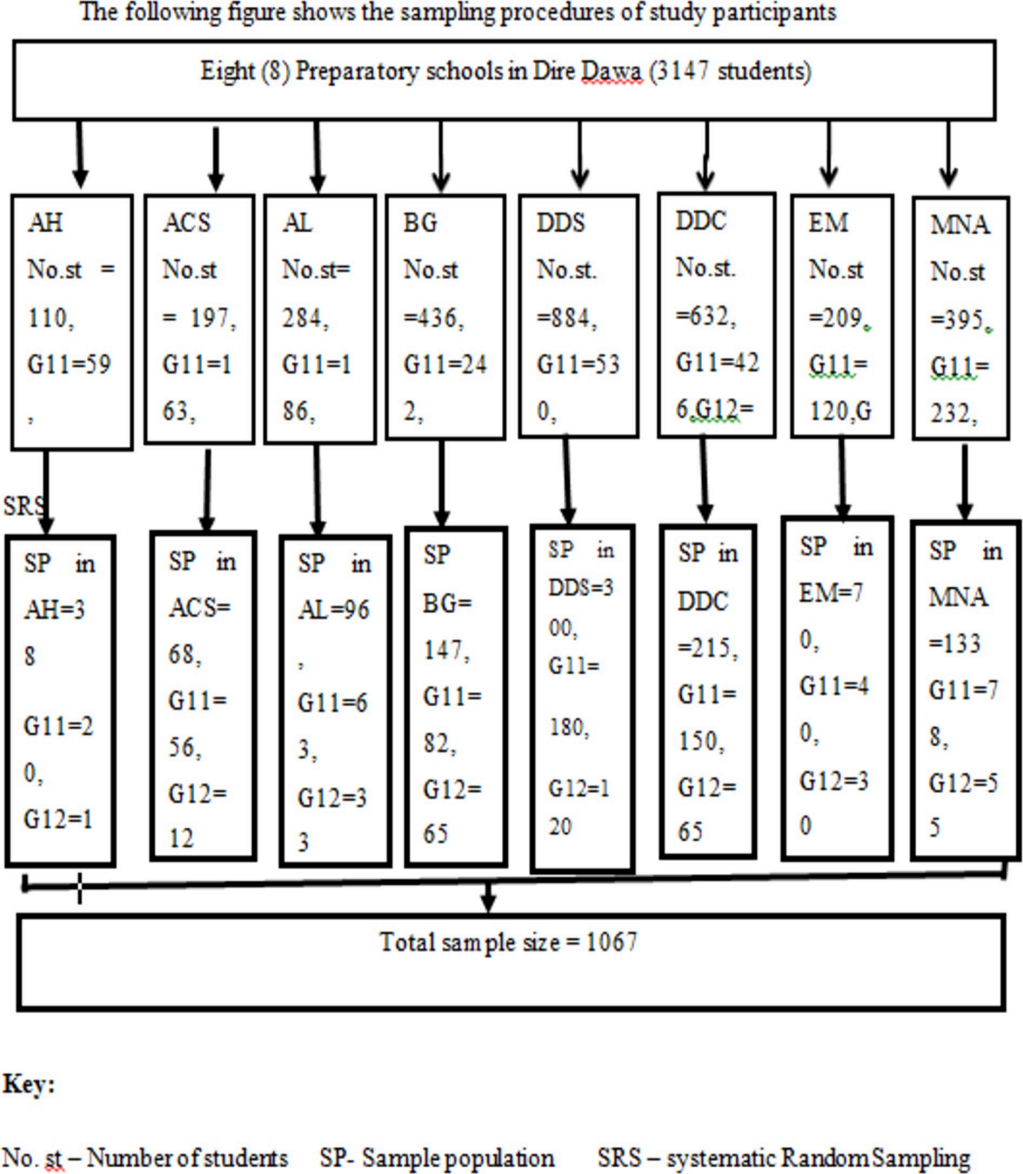
Schematic presentation showing the sampling technique for research on oral and anal sex experience among school youths, Ethiopia. St.no: number of students, SP: sample population and SRS: Systematic Random sampling.

There were a total of 3147 students in eight preparatory schools. The total sample size was proportionally allocated for each preparatory schools and then to grade 11 and 12 according to the number of students. Then systematic random sampling technique was used to select the study participants from each grade (Fig 1)

### Data collection instruments

Data were collected using structured questionnaire adapted from English version [2] and further developed with peer reviewed published literatures.The adapted and the further developed English version questionnaire was translated into Amharic, Afan-Oromo and Somali languages. The questions were both open and closed ended. The tool includes socio demographic variables, sexual experience and related sexual behaviours, anal and oral sex experience, attitude and other contextual factors. The questionnaire was pre-tested in preparatory school in Harar town with 5% of the sample size before the actual data collection. Then the questionnaire was further modified based on identified gaps.

Eight diploma nursing students and Health officers were recruited as data collector and supervisor. Private class room seats were arranged in order to ensure privacy so that one participant couldn’t see others response. Data were collected from all students selected from a particular school simultaneously to overcome information contamination among them. On site supervision was carried out during the whole period of data collection on daily basis by site supervisors and principal investigator.

The Principal Investigator performed both scheduled and unscheduled supervisory visits during the data collection. At the end of each day questionnaires were reviewed and cross checked for completeness, accuracy and consistency by site supervisors and principal investigator and corrective measures were taken. Then data were double entered by two data clerks separately, in to Epi Data and errors related to inconsistency were verified against hard copy of collected data.

### Dependent variables

➢ Oral sex experience
➢ anal sex experience

### Independent variables

Age, sex, grade, ethnicity, maternal educational status, paternal educational status, parental monitoring, best friend anal and oral sex behaviour, alcohol use, chat use, cigarette smoking, shisha smoking and with who he/she live, watching pornography movie, timing of sexual initiation, number of sexual partner, sex with commercial sex worker, consistent condom use, intention for oral/anal sex, attitude towards anal/oral sex, expected outcomes of oral/anal sex.

### Operational definitions

#### Anal sex

“when a man puts his penis in his partner’s anus or when one lets their partner insert the penis in their anus” [2].

#### Oral sex

“when some-one puts his or her mouth on their partner’s penis or vagina or lets their partner put his or her mouth on their penis or vagina [2].

### Data processing and analysis

Cleaned and checked data was entered in to EpiData Version 3.3.1 and exported to SPSS version 20 for analysis. Simple descriptive statistics such as a frequency distribution and percentages was performed. To identify predictors of unusual sexual practices Binary logistic regression with crude odds ratio and its 95% CI was computed. Variables with P < 0.25 in bivariate analysis were entered to multiple logistic regression models to determine predictors of outcome variables. P < 0.05 was considered as a cut off point for statistically significance association. Moreover AOR with their 95% CI would be used to assess strength of the association.

### Ethical considerations

Ethical clearance was obtained from Haramaya University institutional health research ethics and review committee (IHRERC) and permission to collect data was obtained from each schools administration. Informed wwritten and signed consent taken from each study participant. Besides we took assent from their parents or guardians.

## Results

### Socio-demographic characteristics of school youths

A total of 983 (92.1%) response rates were observed. From a total of 983 study participants 513 (52.2%) were male. The mean age of the respondents was 18.63 ± 1.42 SD. About 348 (35.4%) of respondent were Oromo by ethnicity followed by 320 (32.6%) Amhara. A total of 462 (47.0%) were Muslim followed by 433(44.0%) Orthodox Christians. Six hundred twenty four (63.5%) of the participants were living with both biological parents while the remaining 262(36.5%) were in other living arrangements. About 514 (56.1%) of participants mothers were illiterate and had no formal education whereas 650 (68.9%) of participant’s fathers had formal education (Table 2).

**Table 2:** Socio demographic characteristics of study participants in Dire Dawa city administration Eastern Ethiopia 2016.

Variable	Variable category	Frequency	Percent
Age	15-19yrs	732	74.5
	20-24yrs	251	25.5
Ethnicity	Oromo	348	35.4
	Amhara	320	32.5
	Somali	162	16.5
	Others	153	15.6
Religion	Muslim	462	47.0
	Orthodox	433	44.0
	Protestant	79	8.1
	Other	9	.9
Living arrangement	With both biological parents	624	63.5
	With one of the biological parent	210	21.4
	Other living arrangements	52	5.3
Maternal educational status	Illiterate and none formal education	540	56.1
	Formal education	423	43.9
Paternal educational status	Illiterate and none formal education	294	31.1
	Formal education	650	68.9

### Sexual experience of school youths

#### Vaginal sex experience

Two hundred fifteen (22.1%) of the participants had vaginal sex experience out of which 143 (73.7%) started before age of 18. In other way the median age for first sex was17 years with inter quartile range of 3 years. Among those having vaginal sexual experience only 88 (44.4%) were using condoms consistently (Table 3)

**Table 3:** Vaginal sex experience of preparatory school youths in Dire Dawa city administration 2016 (N = 983).

Variable		Number	Percent
Ever engage in vaginal sex	Yes	215	22.1
	No	758	77.9
Age at first sexual initiation	<18 years	143	73.7
	≥18 years	51	26.3
Have sexual intercourse in the last one year	Yes	156	74.3
	No	54	25.7
Sex with commercial sex workers	Yes	26	12.4
	No	183	87.6
Condom use in the last sexual intercourse	Yes	117	56.8
	No	89	43.2
How often do you use condom	Always	88	44.4
	Sometimes	73	36.9
	Never	37	18.7

#### Oral and anal sex experience

About 88(9.0%) had ever engaged in oral sex. Of those who had oral sex experience only 14 (16%) were using condom consistently. While 66(6.7%) had ever engaged in anal sex. Of those who had anal sexual experience only 23(34.8%) were using condom consistently. 44 (65.8%) and 26 (56.5%) school youths had multiple sexual partner for oral and anal sex practice respectively (Table 4)

**Table 4:** Oral and anal sexual experience among preparatory school youths in Dire Dawa city administration, 2016 (N = 983).

Variable		Number	Percent
Ever engaged in oral sex	Yes	88	9.0
	No	895	91.0
Ever engaged in anal sex	Yes	66	6.7
	No	917	93.3
Number of oral sex partner	One	23	34.2
	Two or more	44	65.8
Number of anal sex partner	One	20	43.5
	Two or more	26	56.5
Frequency of condom use during anal sex	Always	23	34.8
	Sometimes	25	37.9
	Never	18	27.3
Frequency of condom use during oral sex	Always	14	15.9
	Sometimes	33	37.5
	Never	41	46.6

### Contextual characteristics of respondents

About 135 (13.8%) and 80(8.2%) participants have intimate partner that practice oral and anal sex respectively. Also 190(20%) of participant’s sexual behavior were monitored by parents. About 234 (24.1%) and 231(23.7%) participants had drinking alcohol and chat chewing experience respectively. About 347 (35.6%) of the participants had ever watched pornography. Related with assessing the participants attitude towards oral and anal sex, 101 (10.7%) and 51(5.6%) of the participants had favorable attitude towards oral and anal sexual practice respectively (Table 5).

**Table 5:** Contextual characteristics of preparatory school youths in Dire Dawa Eastern Ethiopia, 2016 (N = 983).

Variables	Category	Number	Percent
Intimate partner start oral sex	Yes	135	13.8
	No	841	86.2
Intimate partner start anal sex	Yes	80	8.2
	No	890	91.8
Parental monitoring of sexual behavior	Yes	190	20.0
	No	762	80.0
Ever drink alcohol	Yes	234	24.1
	No	735	75.9
Number of bottle of beer per month	1–5	39	27.1
	6–10	51	35.4
	More than 10 bottle	54	37.5
Ever chew chat	Yes	231	23.7
	No	742	76.3
Ever smoke shisha	Yes	115	12.1
	No	837	87.9
Ever watched pornography	Yes	347	35.6
	No	627	64.4
Ever participate in youth and HIV club	Yes	366	37.5
	No	609	62.5
Attitude towards oral sex	Favorable	101	10.7
	Unfavorable	845	89.3
Attitude towards anal sex	Favorable	51	5.6
	Unfavorable	862	94.4

**Fig 2:**
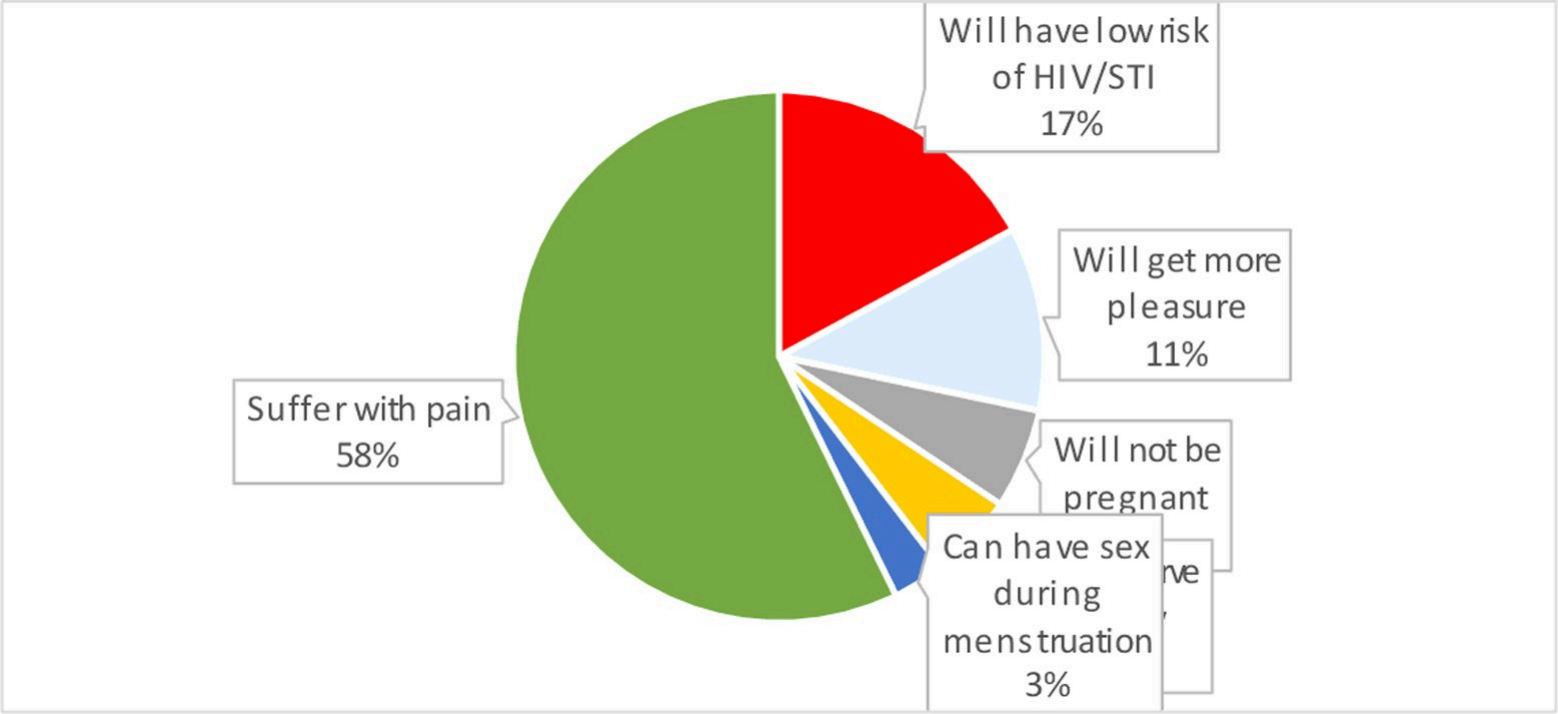
Showing perception of school youths on possible benefits of oral sex among school youths in Dire Dawa, Ethiopia, 2016. Green refers to suffer with pain, Red: have low risk of STI/HIV, Blue one will have more pleasure. (Different colors in the pie chart show the reason/perception of youths for having oral sex).

Concerning to the youths perception on oral sex practice; 32% of the participants perceive that youths having oral sex have low risk of STI/HIV and 29% perceived that youths who practice oral sex can have additional pleasure and other 16% of participants professed that adolescents who practice oral sex could preserve virginity (Fig 2).

**Fig 3:**
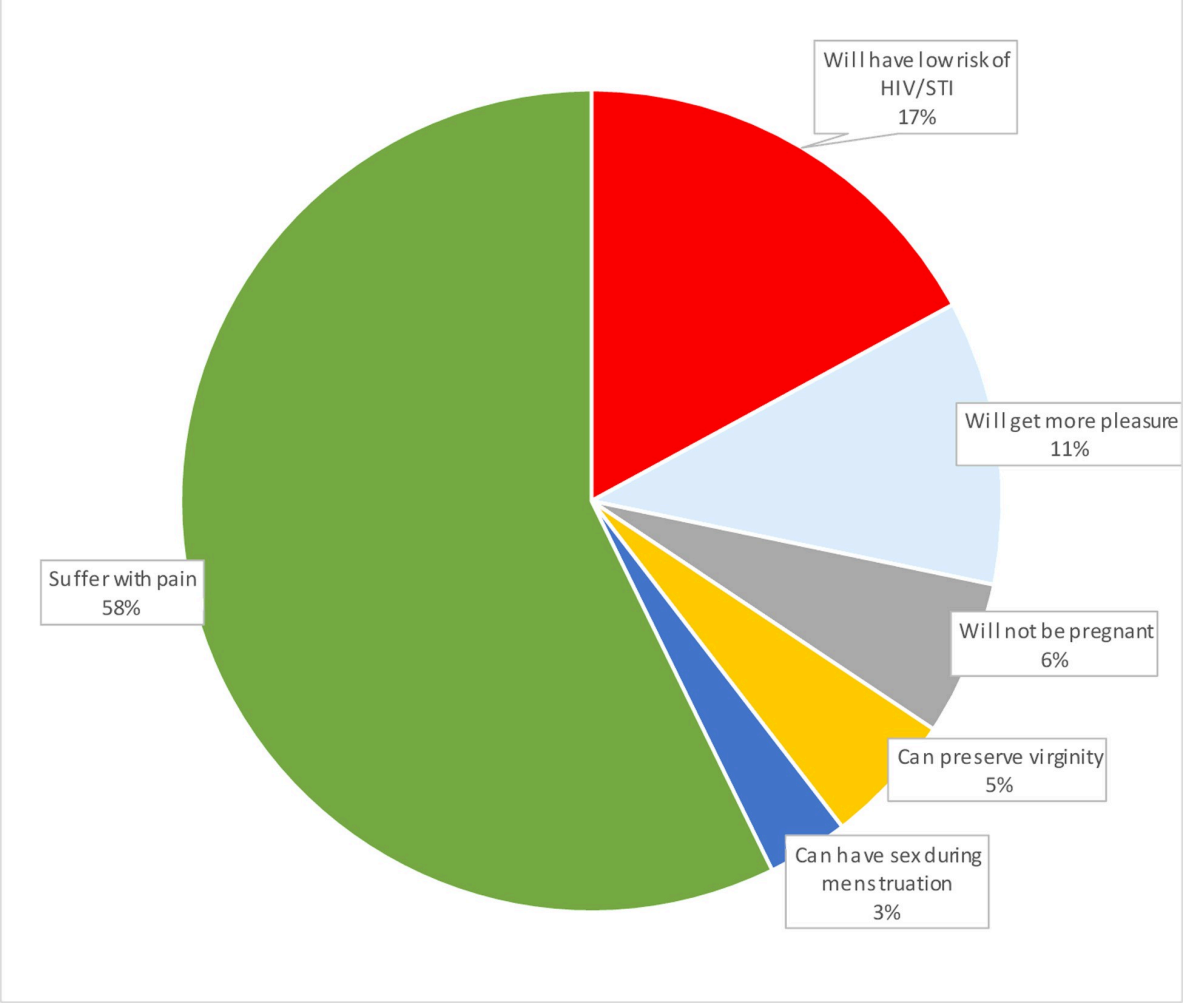
Youths perception on expected outcomes having anal sexual practice among preparatory school youths in Dire Dawa city administration Eastern Ethiopia, March 01-10/2016. Green refers to suffer with pain, Red: have low risk of STI/HIV, Yellow: can preserve virginity: Blue: have more sexual pleasure, Blue black: prevent pregnancy (different colors in the pie chart show the reason/perception of youths for having anal sex).

About 58% of study participants’ perceive that youths who practice anal sex might suffer with pain. The other 17% perceived that youths who practice anal sex might have low risk of STI including HIV (Fig 3).

### Predictors of oral and anal sexual experience

#### Predictors of oral sex among preparatory school youths

As shown in Table 5 below older age (20–24 years) youths have 2.56 times more odds of oral sex experience than younger youths (15–19 years) (COR = 2.56, 95% CI = 1.64–4.01). Those youths whose intimate partner had oral sex experience were 14.87 times more likely had oral sex experience than those whose partner had no oral sex experience (COR = 14.87, 95% CI = 9.13–24.22). School youths who ever drank alcohol were 7.94 times more likely to have oral sex experience than those who didn’t drink ever (COR = 7.94 95% CI = 4.9–12.87). Ever shisha smokers were 7.04 times more odds of oral sex experience than none smokers (COR = 7.04, 95% CI = 4.31–11.45). Youths ever engaged to vaginal sex were 29.46 times more likely to have oral sex experience than their counter parts (COR = 29.46, 15.90–54.59.

On Multivariate analysis, those youths whose age were 20–24 years were 2.45 times more likely to have oral sexual experience than youths whose age were 15–19 years (AOR = 2.45, 95% CI: 1.24–4.86).Among the peer level factors intimate partner oral sex experience was significant predictor for oral sex experience. Youths whose intimate partner had oral sex experience were 4.53 times odds of oral sex experience than their counterparts (AOR = 4.53, 95% CI: 2.26–9.05). Youths who ever drank alcohol were 2.11 times more odds of oral sex experience than youths who didn’t ever drink (AOR = 2.11 95% CI: 1.02–4.34,). Youths who ever smoke shisha were 2.85 times more likely to have oral sex experience than non -smoker (AOR = 2.85 95% CI: 1.40–5.83). Ever engaged in vaginal sex was another strong predictor to oral sex experience. Youths who ever engaged to vaginal sex were 16.38 times more odds of oral sex experience than youths who hadn’t ever engaged vaginal sex (AOR = 16.38, 95% CI: 7.22–37.19) (Table 6).

**Table 6:** Factors associated with oral sex practice among preparatory school youths in Dire Dawa city administration March 01-10/2016 9N = 983).

Variable		Engage in oral sex		COR (95% CI)	AOR (95% CI)
		Yes	No		
Age	15-19yrs	49	683	1	1
	20-24yrs	39	212	2.56(1.64–4.01)	2.45(1.24–4.86)*
Sex	Male	58	455	1.86 (1.18–2.96)	1.21(0.83–3.01)
	Female	30	440	1	1
Educational status	Grade 11	60	517	1.57 (.98–2.50)	1.50 (0.94–4.16)
	Grade 12	28	378	1	1
Type of school	Government	63	517	1.84(1.14–2.98)	1.62 (0.72–2.55)
	Private	25	377	1	1
Living arrangement	With both parents	47	577	1	1
	With one parent	20	190	1.29(.747–2.24)	0.94 (.41–2.14)
	Other living arrangement	10	42	2.92(1.38–6.19)	0.61 (.17–2.18)
Maternal educational status	Illiterate and none formal education	48	492	1	1
	Formal education	40	383	1.07(.69–1.66)	1.31 (.84–2.42)
Paternal educational status	Illiterate and none formal education	33	261	1.45(.92–2.30)	0.95 (0.46–2.73)
	Formal	52	598	1	1
Intimate partner start oral sex	Yes	52	83	14.87(9.13–24.22)	4.53(2.26–9.05)*
	No	34	807	1	1
Monitor by Parent	Yes	23	167	1.64(.99–2.73)	1.97(.90–4.29)
	No	59	703	1	1
drank alcohol	Yes	56	178	7.94(4.90–12.87)	2.11(1.02–4.34)*
	No	28	707	1	1
Ever chew chat	Yes	55	176	6.90(4.35–11.05)	2.18 (.89–10.31)
	No	32	710	1	1
smoke shisha	Yes	35	80	7.04(4.31–11.45)	2.85(1.40–5.83)*
	No	49	788	1	1
watched porno	Yes	66	281	6.46(3.91–10.68)	1.59(.72–3.45)
	No	22	605	1	1
Attitude towards oral sex	Favorable	30	71	5.95(3.59–9.87)	1.26(.57–2.78)
	Unfavorable	56	789	1	1
engaged in vaginal sex	Yes	73	142	29.46(15.9–54.59)	16.38(7.2–37.19)*
	No	13	745	1	1

* shows statistically significant association

#### Predictors of anal sexual experience among school youths

As shown in Table 7, among the peer level factors intimate partner anal sex experience was identified as strong significantly associated predictor variable for youth’s anal sex practice. Youths whose intimate partner had anal sex experience were 20.48 times odds of more anal sex experience than their counterparts (COR = 20.48, 95% CI: 11.58–36.22).

**Table 7:** Factors associated with anal sex experience among preparatory school youths in Dire Dawa city administration 2016.

Variable		Anal sex		COR (95%CI)	AOR(95%CI)
		yes	No		
Age	15-19yrs	38	694	1	1
	20-24yrs	28	223	2.29(1.38–3.82)	1.40(0.66–3.01)
Sex	Male	44	469	1.91(1.13–3.24)	1.54(0.81–3.58)
	Female	22	448	1	1
Educational status	Grade 11	45	532	1.55(0.91–2.65)	2.26(0.79–2.93)
	Grade 12	21	385	1	1
Type of school	Government	48	532	1.93(1.10–3.36)	1.14(0.64–2.39)
	Private	18	384	1	1
Living arrangement	With both biological parents	32	592	1	1
	With one of the parent	17	193	1.63(.89–3.00)	2.06(0.87–4.52)
	Other living arrangements	8	44	3.36(1.46–7.74)	2.29(0.96–6.03)
Maternal educational	Illiterate and none formal	39	501	1.14(.68–1.90)	0.85(0.39–1.72)
	Formal education	27	396	1	1
Paternal education	Illiterate and none formal	27	267	1.68(1.00–2.81)	1.66(0.84–3.02)
	Formal education	37	613	1	1
Intimate partner start anal sex	Yes	34	46	20.48(11.58- 36.22)	5.34(2.39–11.9)*
	No	31	859	1	1
Parental monitoring	Yes	19	171	1.86(1.06–3.27)	2.63(1.12–6.19)*
	No	43	719	1	1
Ever drank alcohol	Yes	40	194	6.68(3.88–11.5)	1.54(.69–3.44)
	No	22	713	1	1
Ever chew chat	Yes	41	190	6.46(3.8–10.95)	3.38(0.98–5.17)
	No	24	718	1	1
Ever smoke shisha	Yes	27	88	6.83(3.9–11.78)	1.79(.80–3.99)
	No	36	801	1	1
Ever watch pornography	Yes	53	294	8.51(4.57–15.9)	3.86(1.5–10.29)*
	No	13	614	1	1
Attitude towards anal sex	Favorable	15	36	7.39(3.78–14.47)	1.38(0.47–4.00)
	Unfavorable	46	816	1	1
Ever engage in vaginal sex	Yes	56	159	33.02(15.44–70.63)	10.64(4.20–26.98)*
	No	8	750	1	1

* shows statistically significant association

Individual level factors were also assessed to determine the predictors of anal sex experience among school youths. Ever drank alcohol (COR = 6.68, 95%CI:3.88–11.51,p), ever chew chat (COR = 6.46, 95%CI = 3.81–10.95), ever smoke shisha (COR = 6.83, 95% CI = 3.96–11.78), ever watched pornographic movies (COR = 8.51, 95% CI = 4.57–15.87), having favorable attitude towards anal sex (COR = 7.39, 95% CI = 3.78–14.47) and ever engaged to vaginal sex (COR = 33.02, 95% CI = 15.44–70.63) were significantly associated with anal sex experience from bivariate analysis.

Among the family level factors living other than biological parents (COR = 3.36 95% CI1.46–7.74) and parental monitoring (COR = 1.86 95% CI = 1.06–3.27) were also significant predictors of anal sex experience.

Youths whose intimate partner engaged to anal sex were 5.34 times more likely to have anal sex experience than those whose intimate partner had no anal sex experience (AOR = 5.34, 95% CI = 2.39–11.9). Parental monitoring were identified as predictor variable for anal sex experience. Youths whose sexual behavior monitored by parents were 2.63 times more likely to have anal sex experience than those who were not monitored (AOR = 2.63, 95% CI = 1.12–6.19).

Ever watched pornographic movies were another individual level factor significantly associated with anal sex experience among school youths. Youths who ever watched pornographic movies were 3.86 times more likely to have anal sex experience than their counter parts (AOR = 3.86 95%CI = 1.49–10.29). Vaginal sex experience was also another predictor variable for anal sex practice for school youths. Youths who ever engaged in vaginal sex were 10.64 times more likely to have anal sex experience than those never engaged vaginal sex (AOR = 10.64, 95%CI = 4.2–26.98) (Table 7)

## Discussion

In this study the proportion of youths engaged in oral and anal sex was 9.0% and 6.7% respectively. This finding is greater than study conducted in Addis Ababa among school youths which was 5.4% oral and 4.3% anal sex practice [1] and very much lower than studies in America and United Kingdom which the proportion of school youths who practice oral sex was 44% and 56% respectively [21]. Similarly, anal sexual experience in this study lower than studies in Brazil and USA Texas which was 43.3% and 18% respectively [22, 23].The variation for this could be the difference in period of study and difference in study area and social setting of the youths.

More over significant proportion of youths practice oral and anal sex without protective measures. Only 16% and 34.8% of youths using condom consistently during oral and anal sex practice respectively; the remaining large proportion of youths practiced oral and anal sexual behaviour without using protection measures. This finding is consistent with previous study conducted in Addis Ababa; only 12.2% and 26.1% youths were using condom consistently during oral and anal sexual practice respectively [1]. The reason might be youth’s low awareness on risk of STI and HIV through such sexual practice. But the risk of acquiring HIV during anal sex practice is 20 times higher than vaginal sex practice[6].Moreover, majority of youths had multiple sexual partner for oral and anal sex practice (65.8% and 56.5% of youths had more than one sexual partner for oral and anal sex practice respectively).

This finding is consistent with the previous study conducted in Addis Ababa (61.2% and 51.1% youths had multiple oral and anal sexual partners respectively) [1]. This indicates that those youths who had oral and anal sexual experience were prone for risk of acquiring STI including HIV. Therefore youths need tailored sexual and reproductive health information in order to make appropriate decision making related to their sexual activity.

The finding of this study shows 22.1% of school youths ever engaged in vaginal sex experience. This finding is comparable with other previous studies, which was engagement to sexual intercourse among school youths ranges 24.1%-67.7% [15, 16, 24]. Even though the proportion of sexually active youths from this study appears lower than previous findings, only 44.4% of them were using condom consistently during sexual activity and 12% of ever engaged in vaginal sex youths, had sex with commercial sex workers. Evidence-based sexual health education programs are effective in decreasing youth sexual risk behaviors, including delaying the onset of sexual activity and increasing the use of condoms among youth who are sexually active [25].

Among the individual level factors older age was significantly associated with youths’ oral sex practice (AOR = 2.45, 95% CI: 1.24–4.86).This finding contradicts with previous study conducted in Addis Ababa Ethiopia [1]. The reason behind might be the difference in age of study participants. The mean age in this study is greater than the previous one. While, youths getting older they attempt to separate from parents and more of stay with peers. Peers become main source of information during this time and had little time to be supervised by parents. In other ways this finding consistent with studies conducted in USA and Brazil [22, 26, 27].

Intimate partner ever engaged in oral sex had strong significance association with oral sex practice (AOR = 4.53, 95%CI: 2.26–9.0). This finding is consistent with previous studies conducted in Addis Ababa, Ethiopia and USA [28]. Best friends usually are main source of information including sexual and reproductive issues. The information that peers discussed may be correct, incorrect, risky information. Such information diffused among peers.

Youth’s substance uses were also another significant predictor for oral sex practice among school youths. Ever drank alcohol and ever smoke shisha were significantly associated independent predictors of oral sex practice among school youths. This finding was supported by other findings in USA [21].

This study showed that there was a strong association between youths who ever engaged in vaginal sexual intercourse with oral sexual experience. Youths who ever engaged in vaginal sexual intercourse were 16.38 times odds of more likely to have oral sex experience than those who hadn’t ever engaged in vaginal sex. This association of ever engaged in vaginal sexual intercourse and oral sexual experience had been found in other studies of adolescents and should be the focus of sexuality education programs aimed at reducing oral sexual behaviors in youths [21, 29].

Youths whose intimate partner practice anal sex were 5.34 times more likely to have anal sexual experience than those whose intimate partner hadn’t anal sex experience (95% CI: 2.39–11.9).This finding is consistent with previous study conducted in Addis Ababa Ethiopia [1]. During youth stage Peers are main source of information rather than families and teachers. Sexual and reproductive issues are among the information shared by youths. Such information wiser good or bad circulates among peers. After a while this age group shares similar characteristics. School based sex education focusing in this social environment is very crucial to diminish risk related to anal sex practice.

Unexpectedly, unlike other study conducted in Addis Ababa [1], anal sexual experience was seen more commonly among youths who had parental monitoring among school youths. This difference in the findings might be due to; the average student age in the previous study was younger than the current sample and younger students might be more likely than older adolescents to have family as more influential of their risk behaviors than peers. In other ways the information that parents provide to their children may not tailored. Even though parents’ monitor the sexual behavior of youths, the information they provide may not address important components of sexuality education. So that the information youths get from peers may divert youth’s decision making.

The odds of youths who had anal sexual experience was higher among youths who ever watched pornographic movies.Youths ever watched pornographic movies were 3.86 times more likely to have anal sexual experience than those youths who hadn’t ever watched pornographic movies (AOR = 3.86, 95%CI:1.45–10.29). Youths require information, and education related with sexuality to identify risks and benefits while they were watching movies.

From this study ever engaged to vaginal sex were significantly associated with anal sexual experience among school youths. Youths ever engaged in vaginal sex had 10.64 times more odds of anal sexual experience than youths who haven’t ever engaged in vaginal sex (AOR = 10.64, 95%:4.20–26.98). This finding is consistent with study conducted in India [30].

Readers of this article need to take the following limitation in consideration while using this article. Since sexual issue in particular oral and anal sex is highly private and sensitive; there might be possibility of underreporting of these sexual behaviors. Moreover this study conducted was based on cross-sectional data, which implies that the direction of association between variables cannot always be determined.

## Conclusions

In general, considerable proportions of youths practiced oral and anal sex, besides majority of them engaged in unprotected sex and with multiple sexual partners while they practiced anal and oral sex. Besides they did not aware STI/HIV transmission risks of anal and oral sex.

Older age, intimate partner ever engaged in oral sex, ever drank alcohol, ever smoke shisha, and ever engaged in vaginal sexual intercourse were identified as independent predictors of oral sex practice.

On the other hand intimate partner ever engaged in anal sex, parental monitoring of youth’s sexual behaviour, ever watched pornographic movies and ever engagement in vaginal intercourse were independent predictors of anal sex practice.

## Recommendation

Though a combination of interventional measures should be invested in order to address and minimize risks related to youth’s sexuality, sexual health education programs need to be designed and promoted to decrease youths sexual risk behaviors, including delaying the onset of sexual activity and increasing the use of condoms among youth who are sexually active. Moreover sexual and reproductive health education and intervention programs which targeted youths need to address anal and oral sexual behavior related issues.

## Abbreviations

AOR: Adjusted Odds Ratio
CDC: Communicable Disease Control
CI: Confidence Interval
COR: Crude Odds Ratio
CSA: central statistical Agency
EDHS: Ethiopian Demographic and Health Survey
HIV/AIDS: Human Immuno deficiency Virus/Acquired Immuno deficiency syndrome
ICF: Action Contre La Faim International
SSA: Sub Saharan Africa
UNAIDS: Joint United Nations Program on HIV/AIDS
